# Taurine: a potential marker of apoptosis in gliomas

**DOI:** 10.1038/sj.bjc.6604933

**Published:** 2009-02-17

**Authors:** K S Opstad, B A Bell, J R Griffiths, F A Howe

**Affiliations:** 1 Division of Basic Medical Sciences, St George's, University of London, Cranmer Terrace, London SW17 0RE, UK; 2 Academic Neurosurgery Unit, St George's, University of London, Cranmer Terrace, London SW17 0RE, UK; 3 Cancer Research UK Cambridge Research Institute, Li Ka Shing Centre, Robinson Way, Cambridge CB2 0RE, UK; 4 Division of Cardiac and Vascular Sciences, St George's, University of London, Cranmer Terrace, London SW17 0RE, UK

**Keywords:** ^1^H MRS, magic angle spinning (MAS), tumour, astrocytoma, apoptosis

## Abstract

New cancer therapies are being developed that trigger tumour apoptosis and an *in vivo* method of apoptotic detection and early treatment response would be of great value. Magnetic resonance spectroscopy (MRS) can determine the tumour biochemical profile *in vivo*, and we have investigated whether a specific spectroscopic signature exists for apoptosis in human astrocytomas. High-resolution magic angle spinning (HRMAS) ^1^H MRS provided detailed ^1^H spectra of brain tumour biopsies for direct correlation with histopathology. Metabolites, mobile lipids and macromolecules were quantified from presaturation HRMAS ^1^H spectra acquired from 41 biopsies of grades II (*n*=8), III (*n*=3) and IV (*n*=30) astrocytomas. Subsequently, TUNEL and H&E staining provided quantification of apoptosis, cell density and necrosis. Taurine was found to significantly correlate with apoptotic cell density (TUNEL) in both non-necrotic (*R*=0.727, *P*=0.003) and necrotic (*R*=0.626, *P*=0.0005) biopsies. However, the ca 2.8 p.p.m. polyunsaturated fatty acid peak, observed in other studies as a marker of apoptosis, correlated only in non-necrotic biopsies (*R*=0.705, *P*<0.005). We suggest that the taurine ^1^H MRS signal in astrocytomas may be a robust apoptotic biomarker that is independent of tumour necrotic status.

Apoptosis is a form of programmed cell death by which an organism can rid itself of damaged or unwanted cells. Neoplastic cells are accumulated by increased cellular proliferation and decreased cellular turnover, and there is much evidence that apoptosis is inhibited in cancer (Kaufmann and Gores, 2000). Such changes are due to multiple genetic aberrations and several have been shown to promote the aggressive characteristics of high-grade tumours (Rao and James, 2004). Dysregulation of apoptosis-regulating proteins in cancer provide targets for drug discovery and new approaches to cancer treatment. If the outcome of a new treatment is to increase apoptosis, a method for monitoring apoptosis *in vivo* for the early detection of drug response would be of great value. One such modality is magnetic resonance spectroscopy (MRS), which can be used to non-invasively determine the biochemical changes in tumour tissue following treatment.

Drug-induced apoptosis studies have shown good correlations between apoptosis and nuclear magnetic resonance (NMR) visible mobile lipids and cytosolic lipid bodies in various human cancer cell lines: DU145 prostatic carcinoma cells (Milkevitch *et al*, 2005), Jurkat T-lymphoblast cells (Di Vito *et al*, 2001; Al-Saffar *et al*, 2002) and HuT 78 lymphoblastoid cells (Iorio *et al*, 2003), suggesting that MRS-detectable lipids accumulate in tumour cells undergoing apoptosis. *In vivo* studies of rodent tumour models have also shown that drug-induced apoptosis results in the accumulation of lipids and specifically polyunsaturated fatty acid (PUFA) resonances at ca. 5.4 and 2.8 p.p.m. (Hakumäki *et al*, 1999; Griffin *et al*, 2003; Schmitz *et al*, 2005). Lipid droplet production appears to occur during both apoptotic and necrotic cell death, and so has been described as a general indicator of cell stress and death (Delikatny *et al*, 2002).

High-resolution magic angle spinning (HRMAS) NMR allows high-resolution spectra to be obtained directly from biopsy tissue, with the added advantage that the same tissue samples can be retrieved post-HRMAS for subsequent histological analysis, thereby allowing a direct comparison between the biochemical and histological profiles (Wu *et al*, 2003; Opstad *et al*, 2008a). In this study, we have used the terminal deoxynucleotidyl transferase biotin-dUTP nick end labelling (TUNEL) method for apoptotic cell detection (Gavrieli *et al*, 1992) in post-HRMAS tissue and compared the estimated metabolite concentrations with the number of TUNEL-positive nuclei per mm^2^ in each biopsy across different grades of glioma. Our aim is to determine a biochemical correlation to apoptosis in gliomas that could provide a robust surrogate marker for apoptosis with *in vivo* MRS.

## Materials and methods

### Tissue collection

Biopsies were collected during routine surgical resection of 41 adult human gliomas, and histology revealed eight astrocytoma grade II (AS2), three astrocytoma grade III (AS3) and 30 glioblastoma (GBM) tumours. All patients were untreated for the tumour, other than dexamethasone, before surgery. Biopsies were snap-frozen in liquid N_2_ and subsequently stored at −80°C. This study was performed with the written informed consent of the entire patients and with the approval from the local ethics committee.

### ^1^H HRMAS spectroscopy

A 10–15 mg sample of biopsy tissue was dissected on cardice and placed in a 50 *μ*l insert (Bruker Biospin Limited, Coventry, UK). The remaining space was then filled with deuterium oxide on ice to maintain the biopsy temperature below 4°C, thus minimising biochemical changes. The insert was then placed in a 4-mm zirconium MAS rotor (Bruker Biospin Limited) and the rotor transferred to the HRMAS probe, which had been precooled to 4°C. Measurements were performed on a 600-MHz Bruker Avance spectrometer, equipped with a quadruple nuclei (^1^H, ^2^H, ^13^C and ^31^P) HRMAS probe, with biopsy samples spun at 5 kHz and maintained at 4°C by a Bruker variable temperature unit. Presaturation spectra were acquired from each biopsy using a repetition time of ≈8 s, with a total acquisition time of 10 min and 57 s. A corresponding spectrum without presaturation was also acquired to obtain a water signal reference.

### Spectral quantification

Each brain tumour biopsy presaturation spectrum was analysed using LCModel (Version 6.1-4) with a presaturation basis set containing 23 metabolite spectra (acetate, alanine, aspartate, choline, creatine, *γ*-aminobutyric acid, glucose, glutamine, glutamate, glutathione, glycine, glycerophosphocholine, lactate, leucine, lysine, *myo*-inositol, N-acetyl aspartate (NAA), NAA-glutamate, phosphocholine, phosphoethanolamine, *scyllo*-inositol, taurine and valine) and 18 simulated lipid/macromolecule (Lip/MM) peaks (Opstad *et al*, 2008b). The metabolite concentrations and Lip/MM proton concentrations were determined using the biopsy water signal as a reference, assuming a water concentration of 44 M (Usenius *et al*, 1994; Cheng *et al*, 1998), with no corrections made for T_1_ or T_2_ relaxation, and therefore all concentrations are expressed semiquantitatively.

### Histology

Following HRMAS, each biopsy sample was removed from its insert, surrounded by optimum cutting temperature (OCT)-embedding compound (Brights Instrument Co., Huntingdon, UK) and refrozen on cardice for cryostat sectioning. Biopsies were sectioned at 10 *μ*m and arranged on polysine microscope slides (VWR International, Lutterworth, UK), so that each slide had five sections representative of the entire length of the biopsy for subsequent analysis.

### Terminal deoxynucleotidyl transferase biotin-dUTP nick end labelling staining

The TUNEL method (Gavrieli *et al*, 1992) was used to stain one slide from each biopsy using a commercially available TUNEL-staining kit (Roche Diagnostics Ltd, Burgess Hill, UK). The first biopsy section on each slide was used as a positive or negative control, as recommended by the manufacturer's instructions. It has previously been reported that TUNEL can label necrotic cells as well as apoptotic cells (Gold *et al*, 1994). However, necrotic areas were clearly visible and showed little, if any, TUNEL-positive nuclei. The TUNEL-positive nuclei were manually counted for the remaining four sections of each biopsy slide using an Olympus BX51 microscope (Olympus Optical Co. Ltd, London, UK). Composite histological images of each section were captured at × 4 magnification, using a digital camera (ColorView 12, Soft Imaging System GmbH, Germany), with the aid of a motorised scanning stage (Prior Scientific Instruments Ltd, Cambridge, UK), and biopsy section areas were determined by analySIS auto software (Soft Imaging System, Munster, Germany). Subsequently, the areas were used to determine the number of TUNEL-positive nuclei per mm^2^ for each biopsy.

### Haematoxylin & eosin (H&E)

Harris' H&E (Sigma-Aldrich, Poole, UK) staining was performed on a consecutive slide per biopsy for morphological determination. Composite histological images were captured at × 10 magnification using the same equipment as before, and the analySIS auto software (Soft Imaging System) used to determine cell density per biopsy by thresholding for the haematoxylin-stained nuclei and expressing as total nuclei per mm^2^. Percentage necrosis was also calculated for each biopsy by measuring the total area of each biopsy section and their necrotic regions of interest.

## Results

**Figure 1:**
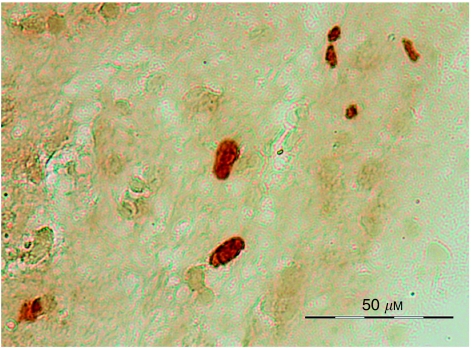
TUNEL-stained nuclei (dark staining) in a post-HRMAS non-necrotic astrocytoma biopsy sample.

Histological analysis of the H&E-stained sections indicated that necrosis was present in 27/41 biopsies (Table 1). For subsequent data analyses, the biopsies were divided into two groups: (i) non-necrotic and (ii) necrotic, according to whether there was a measurable percentage of necrosis. Figure 1 shows TUNEL-staining histology in a post-HRMAS non-necrotic biopsy sample. The TUNEL-positive nuclei are clearly visible (dark stained nuclei) over the background staining.

**Figure 2:**
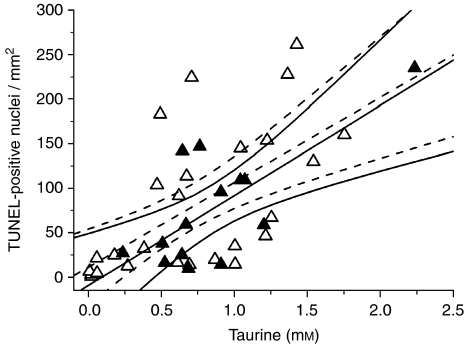
Plots and linear regression analyses (±95% confidence limits) of TUNEL-positive nuclei per mm^2^ against taurine concentration (mM) for non-necrotic (closed symbols and solid lines, *R*=0.727, *P*=0.003, *n*=14) and necrotic astrocytoma biopsies (open symbols and dashed lines, *R*=0.626, *P*=0.0005, *n*=27).

**Figure 3:**
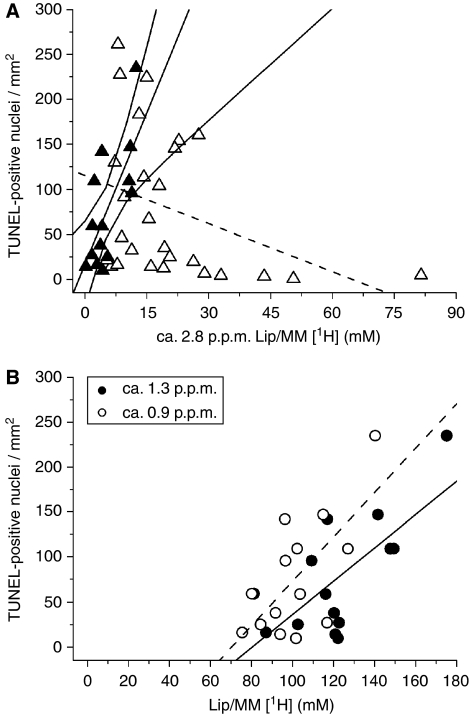
Plots and linear regression analyses (±95% confidence limits) of TUNEL-positive nuclei per mm^2^ against (**A**) ca. 2.8 p.p.m. Lip/MM proton concentration for non-necrotic (closed symbols and solid lines, *R*=0.705, *P*<0.005) and necrotic astrocytoma biopsies (open symbols and dotted line showing the insignificant negative correlation); and (**B**) ca. 1.3 p.p.m. (closed symbols and solid line, *R*=0.703, *P*<0.005) and ca. 0.9 p.p.m. Lip/MM proton concentrations (open symbols and dashed line, *R*=0.676, *P*=0.008) for the non-necrotic biopsies only.

**Figure 4:**
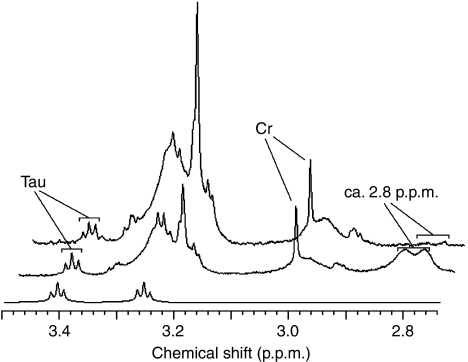
HRMAS ^1^H MRS from a non-necrotic (top) and necrotic (middle) biopsy showing the taurine and ca. 2.8 p.p.m. Lip/MM contributions. The bottom spectrum is an *in vitro* taurine HRMAS ^1^H MR spectrum showing the full taurine spectral pattern. Note that the biopsy spectra are slightly shifted to the right for full view of the spectral peaks.

Linear regression analysis, with Bonferroni correction for multiple comparisons, was used to determine which ^1^H MRS metabolite and lipid concentrations significantly correlated with the apoptotic cell density (TUNEL-stained positive nuclei per mm^2^). Taurine was the only metabolite to significantly correlate with the TUNEL-stained nuclei in both non-necrotic (*R*=0.727, *P*=0.003, *n*=14) and necrotic (*R*=0.626, *P*=0.0005, *n*=27) biopsies, as shown in Figure 2. The slope of the correlation between taurine and the density of apoptotic cells was similar for both groups (non-necrotic and necrotic), with both intercepting very close to the origin. In the non-necrotic biopsies, significant positive correlations were also found between the number of apoptotic cells and the ca. 2.8 and 1.3p.p.m. Lip/MM (Table 2 and Figure 3A and B). A near significant positive correlation was also found between apoptosis and the ca. 0.9 p.p.m. Lip/MM (Table 2 and Figure 3B). However, the necrotic biopsies showed non-significant negative correlations between the apoptotic cell density and the ca. 2.8, 1.3 and 0.9 p.p.m. Lip/MM (Figure 3A for the ca 2.8 p.p.m. Lip/MM; ca.1.3 and 0.9 p.p.m. Lip/MM; data not shown). In the non-necrotic biopsy group, the correlation of apoptotic cell density with the ca. 2.8p.p.m. Lip/MM also intercepted close to the origin (Figure 3A), similar to the correlation with taurine (Figure 2), but this was not true for the correlation of apoptosis with the ca. 1.3 and 0.9 p.p.m. Lip/MM (Figure 3B). Figure 4 shows example for HRMAS spectra of a non-necrotic (top spectrum) and necrotic (middle spectrum) biopsy, highlighting the taurine and ca. 2.8 p.p.m. contributions. The taurine concentrations in both spectra were the same (1.041 and 1.042 mM, respectively), but this figure clearly shows a greater concentration of the ca. 2.8 p.p.m. Lip/MM in the necrotic spectrum (middle spectrum). The percentage of necrosis in this biopsy was 8.8%.

### Cell density and necrosis

**Figure 5:**
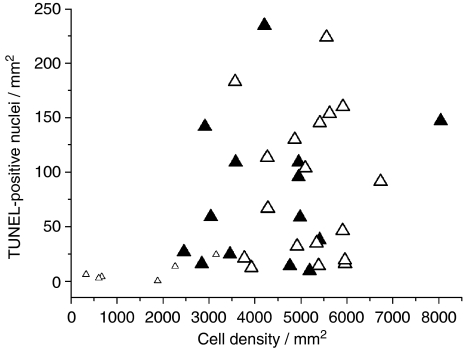
Plots of TUNEL-positive nuclei per mm^2^ against cell density per mm^2^ for non-necrotic (closed symbols, *n*=14), necrotic astrocytoma biopsies with a percentage necrosis <25% (large open symbols, *n*=19) and necrotic astrocytoma biopsies with a percentage necrosis >50% (small open symbols, *n*=6).

Two of the twenty-seven necrotic biopsies had poor H&E staining that prevented accurate measurement of the cell density and percentage of necrosis, so these two specimens were excluded from Figure 5. In Figure 5, the apoptotic cell density was shown to be independent of the total cell density in the non-necrotic biopsies. An apparent correlation of apoptotic cell density with total cell density was found in the necrotic biopsy group (Figure 5), but this was an artefact of the effect of necrotic dilution on both the apoptotic and total cell counts per mm^2^. Similar results were found with the correlation between cell density and taurine concentrations in both biopsy groups (data not shown). Although cell density was found to have a strong negative correlation with the percentage of necrosis in the necrotic biopsy group (*R*=−0.917, *P=*<0.0001, *n*=25), no significant differences were found between the biopsy groups (non-necrotic and necrotic) in cell density, number of TUNEL-positive nuclei per mm^2^ or taurine concentrations. In contrast, the ca. 2.8, 1.3 and 0.9 p.p.m. Lip/MM proton concentrations were all significantly different between the two biopsy groups (*P=*<0.0001, *P=*<0.0001 and *P*=0.01 respectively, Mann–Whitney *U*-test).

## Discussion

The two important findings of this study are (i) taurine concentration in glioma biopsies is correlated with the number of TUNEL-stained apoptotic cells (Figure 2) independently of the presence of necrosis; and (ii) the ca 2.8 p.p.m. Lip/MM peak from PUFAs is correlated with the number of TUNEL-stained apoptotic cells, but only in the non-necrotic biopsies. Thus, we have found two potential biomarkers for the *in vivo* determination of apoptosis in gliomas.

Taurine has been implicated in the mechanism of cell shrinkage during apoptosis in several cell types (cerebellar granule neurons, NIH 3T3 fibroblasts and Jurkat T-lymphocytes (Lang *et al*, 2000; Morán *et al*, 2000; Friis *et al*, 2005)). Cell shrinkage is a distinctive characteristic of apoptotic cells, with changes in ion channel fluxes thought to play a major part (Gómez-Angelats and Cidlowski, 2002). However, the exact mechanism of this is unclear. One study has implicated p38 mitogen-activated protein kinase activation before an efflux of potassium and taurine from the cell, resulting in stimulation of the cysteine protease caspase-3 in NIH 3T3 fibroblasts (Friis *et al*, 2005). Another study has implied that CD95-receptor triggering leads to a caspase-dependent stimulation of cellular taurine release, thereby facilitating apoptotic DNA fragmentation (labelled by the TUNEL reaction (Watanabe *et al*, 2002)) and cell shrinkage in Jurkat T-lymphocytes (Lang *et al*, 2000). Whether taurine accumulation occurs only in relation to apoptotic cells, or whether apoptosis and increased taurine content derive from a common cause such as hypoxia, resulting in a population of cells with increased taurine of which some are in the apoptotic stage, is as yet unknown. Further study is now needed to understand the processes that give rise to the correlation between apoptosis and taurine in gliomas as shown in Figure 2.

Necrosis is one of the histopathological indicators of malignancy in astrocytomas, and Table 1 highlights a discrepancy in a few tumours between their clinical grading and the presence of necrosis in the HRMAS biopsy specimens used in this study. This is likely a result of sampling error and the heterogeneity of astrocytomas, and does not affect the interpretation of our results that show direct correlation of the HRMAS data with histopathological analysis of the same piece of tissue. The presence of significant amounts of necrosis (or large variations in cell density) can also produce false correlations between any two parameters whose value is dependent on the number of viable cells. In Figure 5, the number of TUNEL-positive nuclei per mm^2^ is shown to correlate with the total biopsy cell density when all the necrotic biopsies were included in the analysis, but this was because of the six biopsies (plotted as small open triangles) that had a percentage necrosis of greater than 50%, and hence very low TUNEL-staining. However, by treating those samples as outliers and comparing only the samples with <25% necrosis, it can be seen that there is no correlation between the number of TUNEL-positive cells per biopsy sample and cell density. Thus, our result of a correlation between taurine and TUNEL-staining is a true correlation independent of cell density or necrosis, when the highly necrotic samples are excluded.

The ca. 2.8 p.p.m. PUFA ^1^H MRS lipid peak has been previously assigned as a biomarker of apoptotic response in a study of anticancer treatment by gene therapy in preclinical rodent gliomas (Hakumäki *et al*, 1999; Griffin *et al*, 2003) and also proposed as an *in vivo* biomarker for apoptosis in the clinic (Hakumäki *et al*, 1999; Schmitz *et al*, 2005). We also observed a good correlation between the ca. 2.8 p.p.m. (-CH=CHCH_2_CH=CH-) PUFA proton concentrations and the number of TUNEL-stained apoptotic nuclei in human glial brain tumour biopsies, but only in the non-necrotic biopsies with an intercept close to the origin (Table 2 and Figure 3A). This suggests that the PUFA peak at ca. 2.8 p.p.m. originates during the process of apoptosis before necrosis (Hakumäki *et al*, 1999; Griffin *et al*, 2003). Correlations were also found between apoptosis and the ca. 1.3 p.p.m. (-CH_2_-) and ca. 0.9 p.p.m. (-CH_3_) Lip/MM resonances in the non-necrotic biopsies (Table 2 and Figure 3B), but the non-zero intercept indicates the presence of additional ca. 1.3 and 0.9 p.p.m. signals that do not relate to apoptosis.

It has been suggested that apoptosis produces lipid bodies that remain after cell death (Griffin *et al*, 2003), similar to those observed with necrosis in rodent gliomas (Rémy *et al*, 1997; Lahrech *et al*, 2001; Zoula *et al*, 2003). However, in the necrotic biopsies, we observed a correlation between the percentage of necrosis and the ca. 2.8 p.p.m. proton concentration (*R*=0.663, *P*=0.0003, *n*=25, data not shown), but no correlation was found between the ca. 2.8 p.p.m. peak and the number of TUNEL-positive nuclei. This suggests that the ca. 2.8 p.p.m. resonance in the necrotic biopsies arises primarily from hypoxic/necrotic lipid bodies rather than apoptotic lipid bodies. Overall, our data are in agreement with earlier findings in animal studies that the PUFA peak at ca. 2.8 p.p.m. originates during the process of apoptosis, but our results (Figure 3A) suggest that this correlation is only true for low-grade astrocytomas, which are non-necrotic.

A recent pattern recognition of HRMAS study of cervical carcinoma biopsies (all except one being non-necrotic) has shown that all the lipid peaks in the presaturation spectra contributed to the principal components that correlated with the apoptotic cell density, with a major contribution from the ratio of the ca. 1.3 p.p.m. to ca. 0.9 p.p.m. Lip/MM peaks. Taurine was found to be unrelated to apoptosis in these cervical carcinoma biopsies, but instead related to the tumour cell density and tumour fraction when combined with creatine, choline-containing metabolites, glucose and lactate (Lyng *et al*, 2007). In our study, we did not find any correlations between apoptosis (either the number of TUNEL-positive nuclei per mm^2^ or the apoptotic index (calculated as the percentage of TUNEL-positive nuclei per cell density)), and the ca. 1.3 p.p.m.:ca. 0.9 p.p.m. Lip/MM ratios. Therefore, it appears that biomarkers associated with apoptosis may be dependent on cancer type.

Taurine concentrations have been shown to remain stable for up to 8 h in postmortem human brain (Perry *et al*, 1981), and we have also recently shown taurine to remain stable in human brain tumour biopsies (AS2 and GBM) and normal rat brain during prolonged HRMAS spinning (Opstad *et al*, 2008c). Thus, significant postischaemic changes in tumour biopsy taurine concentrations as a result of the biopsy excision, and/or the HRMAS procedure appear unlikely. Taurine is generally considered difficult to accurately measure in *in vivo* ^1^H MRS because of signal overlap from the stronger signals of *myo*-inositol and the cholines at ≈3.2 p.p.m. and glucose at ≈3.4 p.p.m. (Govindaraju *et al*, 2000), with an exception being the observation of highly elevated taurine in medulloblastomas (Moreno-Torres *et al*, 2004). Generally, we would expect taurine editing, which has previously been applied to *in vivo* ^1^H MRS of normal rat brain (Lei and Peeling, 1999), would provide a more robust measurement of taurine, and hence the use of taurine as an *in vivo* biomarker of apoptosis (both pre- and posttherapeutic responses) for differing grades of astrocytoma may be possible in the clinic.

In conclusion, we have shown that the taurine concentration in astrocytomas correlates with the number of TUNEL-stained apoptotic nuclei independently of the presence of necrosis. Our data also suggest that taurine may be a better biomarker of apoptosis in glial tumours than the ca 2.8 p.p.m. PUFA peak, for which a correlation with apoptosis is only found in non-necrotic biopsies. These conclusions suggest that the measurement of taurine in gliomas *in vivo* by non-invasive MRS could be a useful technique for monitoring tumour apoptosis in the clinic.

## Figures and Tables

**Table 1:** Relationship between the necrotic status of the individual HRMAS tumour biopsies and the grade of the originating tumour as histopathologically diagnosed from multiple biopsies during surgical excision

	**Diagnosed grade of originating tumour**			
**Biopsy status**	**AS2**	**AS3**	**GBM**	**Total**
Non-necrotic	7	2	5	14
Necrotic	1	1	25	27
Total	8	3	30	41

**Table 2:** Correlations of HRMAS ^1^H MRS metabolite and lipid concentrations with TUNEL-positive nuclei per mm^2^ for non-necrotic astrocytoma biopsies

**Metabolite**	***R***	***P*-value**
Tau	0.727	0.003^*^
ca. 2.8 p.p.m.	0.705	<0.005^*^
ca. 1.3 p.p.m.	0.703	<0.005^*^
ca. 0.9 p.p.m.	0.676	0.008

^*^Correlations are significant at *P*=0.005 after Bonferroni correction for multiple comparisons.
